# Microbiological Surveillance and Antimicrobial Susceptibility Observations on Peritoneal Dialysis-Associated Peritonitis in an Outpatient German Reference Center

**DOI:** 10.3390/idr17030049

**Published:** 2025-05-03

**Authors:** Annemarie Albert, Stefan Richter, Lisa C. Costello-Boerrigter, Philipp Stieger, Rainer Peter Woitas, Rüdiger C. Braun-Dullaeus, Christian Albert

**Affiliations:** 1 Diaverum Renal Services, Am Neuen Garten 11, 14469 Potsdam, Germanyrainer.woitas@diaverum.com (R.P.W.); 2 Department of Nephrology, Central Clinic Bad Berka, Robert-Koch-Allee 9, 99438 Bad Berka, Germany; 3 Department of Cardiology and Intensive Care Medicine, Central Clinic Bad Berka, Robert-Koch-Allee 9, 99438 Bad Berka, Germany; 4 University Clinic for Cardiology and Angiology, Otto-von-Guericke University Magdeburg, Leipziger Str. 44, 39120 Magdeburg, Germany

**Keywords:** peritoneal dialysis, peritonitis, Gram-negative organisms, Gram-positive organisms, antibiotic stewardship, antibiotics, renal replacement therapy, pathogen spectrum

## Abstract

**Background**: Peritonitis is a relevant complication in peritoneal dialysis (PD). The initial empirical antibiotic therapy depends on the center-specific distribution of microorganisms and the microbial susceptibility profiles. However, data on the locoregional germ spectrum in Germany are insufficient regarding the current recommended empirical antibiotic regimens of either cefepime as monotherapy or the combination of cefazolin and ceftazidime. **Methods**: This retrospective single-center study of routine clinical patient data analyzes the range of infecting organisms causing PD-associated peritonitis and their corresponding antimicrobial resistances during the 2015 to 2022 timeframe. We used Ordinary Least-Squares regression to model trends in the detection of microbiological spectrum samples. The ‘reporting of studies conducted using observational routinely collected health data’ (RECORD) statement was acknowledged. **Results**: There were 80 documented peritonitis episodes with 99 causal etiologies sampled. Of those, eighty-seven were bacterial, three were fungi (3%), eight had no microbial growth (8%), and one more had missing data. The largest group of microorganisms detected were Gram-positive bacteria (N = 56, 56.6%), predominantly sampled as Staphylococcacea, Enterococcaceae, and Streptococcaceae (*Staphylococcus aureus*, 14.1%). Gram-negative bacteria were found in 31.3% of samples (N = 31), predominantly Enterobacteriaceae (*Escherichia coli*, 9%). In total, 34 different microorganisms were identified. On one occasion, methicillin-resistant *Staphylococcus epidermidis* and one sample of multi-resistant *Serratia marcescens* were identified. Methicillin-resistant *Staphylococcus aureus* and vancomycin-resistant enterococci were not detected. Fungi were found in three peritonitis episodes. Regression analyses did not indicate changes in the general microbiological spectrum during the observational timeframe. The center-specific peritonitis rates were below the recommended rates of the International Society for Peritoneal Dialysis for all years studied. **Conclusions**: The recommended empiric therapy was suitable at our center, with a few exceptions for non-specific pathogens and for those with β-lactamases or enterococci. When there is no clinical response to empiric therapy, alternative antibiotics should be considered accordingly. The retrospective data are limited to the reported outcome measures.

## 1. Background

Peritoneal dialysis (PD) is a widely used renal replacement therapy that allows end-stage renal disease patients to have home-based treatment [1]. However, persons undergoing PD independently at home are occasionally faced with relevant medical or procedural complications [2]. Specifically, the connect and disconnect procedures are associated with the risk of contamination with subsequent development of PD-associated peritonitis [3].

Excluding pre-PD peritonitis and PD catheter insertion-related peritonitis, the risk of PD-associated peritonitis is 0.26–0.40 episodes per patient per year (peritonitis rate) worldwide [4]. To increase further the adoption of PD in an ambulatory center, it is therefore mandatory to address this complication adequately. The 2022 guidelines from the International Society for Peritoneal Dialysis (ISPD) recommend that dialysis programs monitor yearly peritonitis rates with a goal peritonitis rate of 0.40 or less [4]. It is likewise recommended that organism-specific peritonitis rates, antimicrobial susceptibilities, and culture-negative peritonitis all be monitored and reported [4]. In general, PD-associated peritonitis can be successfully treated with appropriate intraperitoneal (IP) antibiotics.

However, currently knowledge of the locoregional microbiological spectrum in Germany is insufficient. Therefore, in keeping with the ISPD recommendations and guiding the implementation of an antibiotic stewardship program to improve empirical therapy and to monitor our center’s resistance rates in PD-associated peritonitis [5], we report for the first time the range of infecting organisms causing PD-associated peritonitis and their corresponding antimicrobial resistances in our representative PD cohort from northeastern Germany.

## 2. Materials and Methods

### 2.1. Data Collection

Clinical data were retrospectively collected from patients undergoing PD at the Diaverum reference center in Potsdam, Germany. The data covered the time period from 1 January 2015 to 31 December 2022 and included patient numbers and characteristics, PD dates, PD modality, peritonitis date, causative microorganisms, bacterial sampling, antibiotic sensitivity testing for medication(s) used. Additional yearly data, such as total delivered PD treatment days and total number of patients, were extracted to calculate the center-specific peritonitis rate.

### 2.2. Diagnosis

PD-associated peritonitis was defined according to the ISPD 2022 guidelines, which require the presence of two of the following three findings: clinical features consistent with peritonitis (e.g., abdominal pain, cloudy dialysis effluent), dialysis effluent white blood cell (WBC) count > 100/µL or >0.1 × 10^9^/L (after a dwell time of at least 2 h) with >50% polymorphonuclear leukocytes (PMN), and/or a positive dialysis effluent culture [4].

The definition of a medical cure was likewise based upon ISPD 2022 guidelines and consisted of the complete resolution of peritonitis and no relapse, recurrent peritonitis, catheter removal, transfer to hemodialysis for ≥30 days, or death [4].

### 2.3. Standardized Sample Handling

Under sterile conditions, a volume of 20 mL dialysate was collected and placed in a pair of aerobic and anaerobic blood culture bottles for testing. If patients presented to our center with a dry cavity, then according to ISPD recommendations, a dwell for one hour was performed before using the drained dialysate for analysis. If the patient noticed cloudy dialysate from the prior night’s dwell, then the patient was instructed to bring the night bag to our center for diagnostics.

All samples were collected twice daily from our center by our associated service partner Labor 28 (Berlin, Germany), which specializes in laboratory medicine and microbiology. Gram staining was performed according to protocol. All cultures were grown, tested, and analyzed for antimicrobial sensitivities by Labor 28 (Berlin, Germany) according to the respective manufacturer’s recommendations. Blood culture bottles (BACTEC) were used from BD (Becton Dickinson and Company, Heidelberg, Germany) and were routinely incubated for seven days. Bacterial identification was performed using the MALDI Biotyper System (Bruker Corporation, Billerica, MA, USA).

### 2.4. Evaluation of Microbial Spectrum and Resistance Profile

To determine the microbial spectrum for the region, all causative microorganisms were recorded and grouped according to species, microbiological staining behavior (Gram stain), and morphology. It was also recorded if no microorganism could be identified (culture-negative peritonitis) or if patient data were missing.

To analyze the resistance profiles, the causative organisms were classified according to three main groups (Gram-positive, Gram-negative, and fungi) and, when possible, according to family and species type. Multi-resistant pathogens were reported separately.

### 2.5. Treatment Regimens

At the Diaverum dialysis center, antibiotic treatment was performed IP at initial patient presentation and continued daily in the long interval exchange using an icodextrin solution (Baxter, BX, Unterschleißheim, Germany) with a dwell time of at least six hours. Cefazolin and ceftazidime were administered as the daily empirical antibiotic therapy until 2019. Since January 2019, cefepime was used as monotherapy. Both selections are consistent with the ISPD’s 2022 guidelines, which suggest the use of a first-generation cephalosporin or vancomycin to cover Gram-positive organisms and a third-generation cephalosporin or an aminoglycoside to cover Gram negatives or, alternatively, cefepime monotherapy [4]. The antibiotic regimen was then refined after identification of the causative organism(s) and their respective antimicrobial resistances. The duration of therapy was dependent upon the pathogen.

Given that additional antibiotic regimens were available at the Diaverum center and their collaborating hospitals, those antibiotics were also included in the summary resistance analysis where reasonable and where the data were available.

### 2.6. Ethical Approval

This retrospective analysis of routinely collected clinical patient record data was part of an internal quality improvement program. Given the retrolective nature of this investigation, necessity for patient consent to participate was exempt by the institutional review board of Diaverum Germany in accordance with German legal requirements (§ 27 BDSG, Bundesdatenschutzgesetz) enabling anonymized ‘data processing for scientific or historical research purposes’. The study protocol was in concordance with the ethical guidelines of the Declaration of Helsinki and was approved by both the Institutional Review Board of Diaverum and the Otto-von-Guericke University Medical Faculty Ethics Committee, Magdeburg, Germany, Case No. 142/22 (approved on 21 November 2022).

### 2.7. Analysis

The center-specific peritonitis rate (PR) was calculated using Formula (1) for all years of the study period [6].

(1) PR=Sum of Peritonitis Episodesyear for N Pts.∑Pt.1Pt.N∑PD daysyear365.25

The conduction of the study conformed to the ‘reporting of studies conducted using observational routinely collected health data’ (RECORD) statement [7].

We used two methods to estimate temporal trends in the detection of microbiological spectrum samples. First, we applied Locally Estimated Scatterplot Smoothing (LOESS) to provide an estimate representation of non-linear detection rate trends over time by fitting local polynomial regressions to subsets of the data. To illustrate the uncertainty around the smoothed curves, the associated confidence areas were computed using standard error estimates from the LOESS fit.

As a secondary approach, an Ordinary Least-Squares (OLS) regression model was applied separately for each category of microorganisms. The detection rate was modeled as a function of time (years, 2015 to 2022), where the slope coefficient (β1) indicated the direction of the trend. To determine the statistical significance of these trends, a *p*-value was obtained from the regression model and considered significant when below 0.05. We calculated the 95% confidence intervals for the linear regression using the standard error of the estimated regression coefficients.

All statistical analyses and illustrations were performed using the R environment for statistical computing (R Foundation for Statistical Computing, Vienna, Austria) [8] with the *mass* package version 7.3-64 [9].

## 3. Results

### 3.1. Demographic and Clinical Characteristics

From 1 January 2015 to 31 December 2022, the dialysis practice being evaluated cared for 129 patients who were undergoing or initiating PD. The most frequent PD procedure performed was automated PD (APD) for 79 patients. Continuous ambulatory PD (CAPD) was performed in 39 patients and intermittent PD (IPD) in 11 patients. The median time from PD initiation to first peritonitis episode was 13 months (Table 1).

### 3.2. Distribution of Peritonitis Episodes

Forty-six patients (35.66%) were affected by at least one episode of peritonitis, with a total of 80 episodes occurring in the observational timeframe, while 83 (64.34%) had no peritonitis episodes.

**Figure 1:**
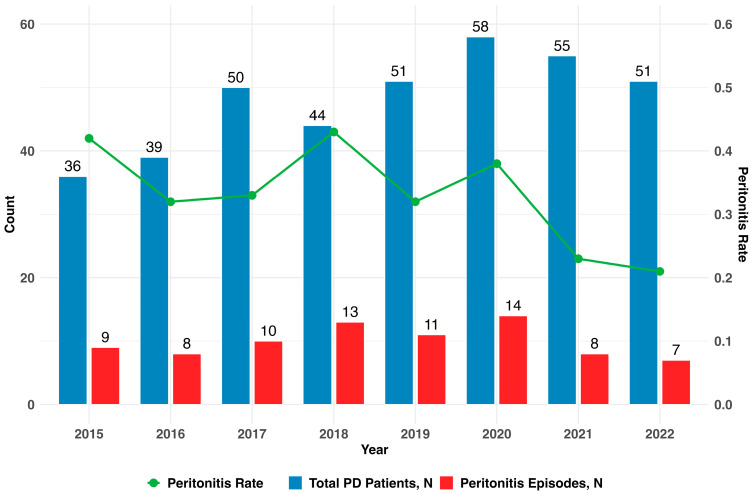
Assessed number of PD patients, peritonitis episodes, and the corresponding peritonitis rate over time. As suggested by the ISPD in 2022, the overall peritonitis rate should be no more than 0.40 (down from 0.5 before that).

The number of patients receiving PD increased over time, from 36 in 2015 to 51 in 2022, with a peak of 58 in 2020. The number of peritonitis episodes likewise peaked in 2020 (N = 14); however, despite the increase in patients from the beginning to the end of the period, the relative number of peritonitis episodes as calculated by the center-specific peritonitis rate declined from 2015 to 2022 as shown in Figure 1.

### 3.3. Spectrum of Causative Bacteria

In total, we detected 87 bacterial microorganisms. The largest group of microorganisms detected were Gram-positive bacteria (N = 56), followed by Gram-negative bacteria (N = 31). Fungi (*Candida* spp.) formed the smallest etiological group being detected in three peritonitis episodes. In total, 34 different microorganism were identified (Table 2, Supplementary Figure S1). *Staphylococcus* spp., predominantly *Staphylococcus aureus* and *Streptococcus* spp. were the most commonly identified Gram-positive bacteria. In the Gram-negative spectrum, *Escherichia coli* and *Enterobacter cloacae* were the most frequently detected pathogens. Other organisms identified were all fungi from the *Candida* spp.

**Figure 2:**
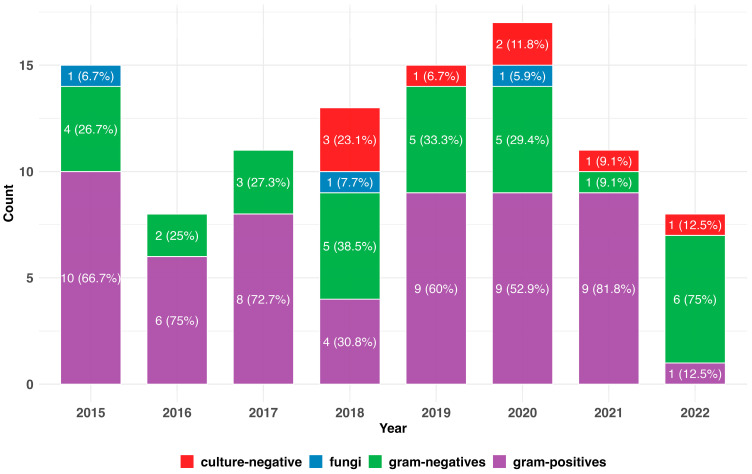
Overview of the pathogenic spectrum found each year over the 2015–2022 period, N = 98.

The ISPD guidelines recommend that the methods used for sampling and incubation be reviewed if microbial growth is not possible in more than 15% of cases [4]. Overall, there were culture-negative samples on eight occasions during the period under review (8.08%). However, on a per year basis, the number of culture-negative tests exceeded the ISPD recommendation in 2018 at 23.1% (N = 3). One peritonitis episode had missing data (Figure 2).

### 3.4. Changes in the Pathogenic Spectrum over Time

Gram-positive bacteria were the most common pathogenic organisms every year, except for 2018 and 2022, when Gram-negative bacteria surpassed them. The difference between Gram-positive and Gram-negative pathogens was greatest in 2021, although the numbers were small (Supplementary Table S1).

Fungi were only identified in three years (2015, 2018, and 2020), with only one detection in each of those years (Figure 2). There were multiple episodes with sampling of multiple organisms. Both were associated with at least temporal peritonitis associated modality switch to hemodialysis (Supplementary Table S2).

**Figure 3:**
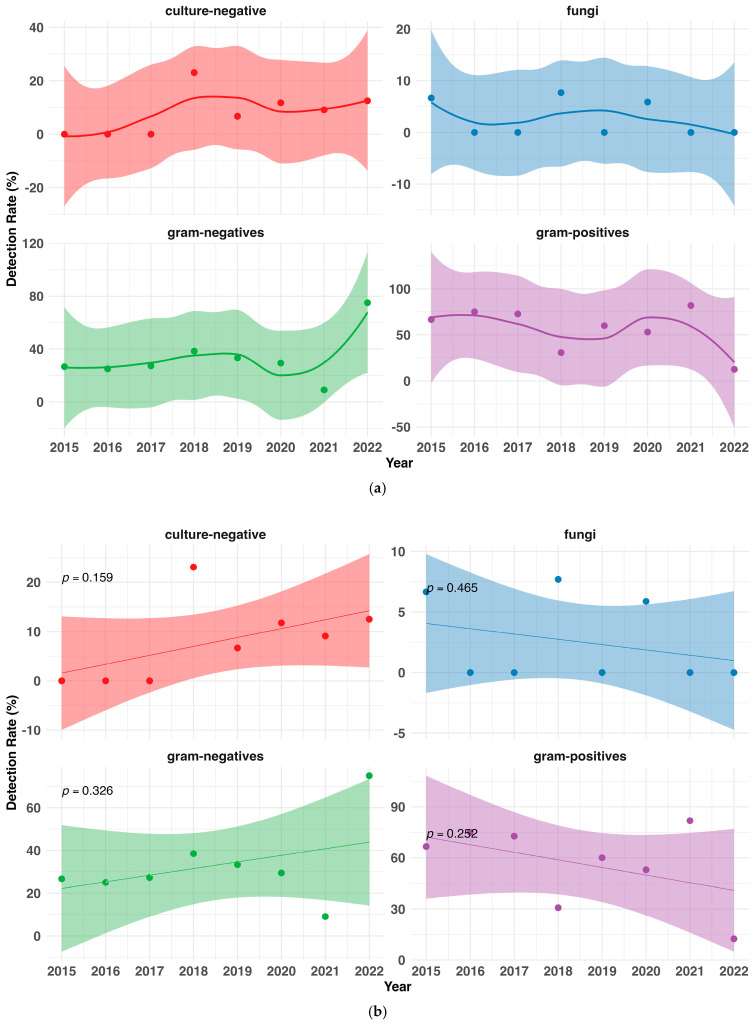
(**a**) Locally Estimated Scatterplot Smoothing (LOESS) was applied to local polynomial regressions to model detection rate trends in microorganism categories over time to provide a visual estimate of non-linear trends. (**b**) Microorganism detection rates per year with linear trend estimates, calculated using ordinary least-squares regression (OLS) with 95% confidence intervals.

However, in the observational timeframe, there were no significant trends regarding the sampling of Gram-negative (*p* = 0.326) or Gram-positive (*p* = 0.252) pathogens, fungi (*p* = 0.465), or frequency of culture-negative samples (*p* = 0.159) (Figure 3a,b, Supplementary Figure S2).

In Supplemental Figure S3, we explored the hypothesis that the higher Gram-negative count, as opposed to the expected Gram-positive count seen in Figure 2 in the last study year, might be an outlier phenomenon and excluded the year 2022 from the data assessment. Following this hypothesis, and excluding the 2022 data, there was no indication of an increase in Gram-negative bacteria (*p* = 0.485) and an almost horizontal trend line for the Gram-positive bacteria (*p* = 0.913).

### 3.5. Resistance Profiles

The available sensitivity testing results for Gram-positive and Gram-negative bacteria, respectively, are summarized in Table 3. Out of 90 differentiated microorganisms, there were 29 resistance profiles missing (of 22 individual peritonitis episodes). *Staphylococcus aureus*, one of the four most commonly detected Gram-positive bacteria were found to be completely sensitive to the primary antibiotics recommended by the Paul Ehrlich Society guidelines for intravenous therapy of PD-associated peritonitis [10], namely cefazoline, cefuroxime, ceftriaxone, ceftazidime, and ciprofloxacin. The *Streptococcus* spp. behaved similarly, with the exception of resistance to ciprofloxacin.

The present analysis primarily focused on antibiotics outlined in the ISPD guidelines for IP treatment, in particular cefazolin, cefepime, vancomycin, and gentamicin. *Staphylococcus aureus*, *Strepotococcus* spp., and *Enterococcus* spp. only showed resistance to gentamicin. *Staphylococcus epidermidis*, on the other hand, demonstrated resistance to cefazolin and cefepime. Additionally, oxacillin resistance was noted in four other Gram-positive samples of *Staphylococcus epidermidis*, *Streptococcus salivarius*, *Staphylococcus hominis*, and *Listeria monozytogenes*. *Enterococcus* spp. showed the highest distribution of resistance to all cephalosporin antibiotics tested, which was expected given their known intrinsic resistance to antibiotics that are directed against the bacterial cell wall [11]. Ciprofloxacin was the only Paul Ehrlich Society recommended antibiotic to which these species were not resistant.

In the group of Gram-negative bacteria, *Enterobacter cloacae*, and *Klebsiella* spp. were sensitive to the primary, recommended antibiotics. *Escherichia coli* was only consistently sensitive to cetriaxone. Yet, the Gram-negative group of pathogens showed full sensitivity to the ISPD antibiotics tested.

Other antibiotics analyzed for sensitivity were linezolid, which is used as a reserve antibiotic for Gram-positive bacteria, penicillin G for streptococcal infections, ampicillin/sulbactam, and piperacillin/tazobactam for an extended spectrum of activity against Gram-positive and Gram-negative bacteria, and imipenem and piperacillin/tazobactam against *Pseudomonas aeruginosa*. Gram-positive pathogens showed full sensitivity to linezolid and streptococci to penicillin G. *Staphylococcus epidermidis* and *Enterococcus* spp. showed resistance to ampicillin/sulbactam, piperacillin/tazobactam, and imipenem. In the group of Gram-negative pathogens, a sensitivity to the same antibiotics was observed with only ampicillin/sulbactam standing out as an exception.

### 3.6. Multi-Drug-Resistant and Atypical Pathogens

In the group of staphylococcaceae, we found resistance to penicillin G and ampicillin, which is rather expected; however, one sample identified *Staphylococcus epidermidis* was resistant to oxacillin fulfilling MRSE criteria. Methicillin-resistant *Staphylococcus aureus* (MRSA) and vancomycin-resistant enterococci (VRE) were not detected. Multi-resistant Gram-negative pathogens (MRGN) were detected in one sample of *Serratia marcescens* that was resistant to four classes of Gram-negative-active antibiotics (*4MRGN*) (Table 3). There were no 3MRGN sampled.

The sampled *Candida* spp. were sensitive to all major antifungal medication (amphotericin, caspofungin, fluconazol, micafungin, and voriconazol).

**Table 3:** In vitro susceptibility rates of selected Gram-positive and Gram-negative pathogens with N > 2 identified samples.

Antibiotics	Microorganisms [s (r)]								
	*Staphylococcus aureus*	*Staphylococcus epidemidis*	*Streptococcus* spp.	*Enterococcus* spp.	*Enterobacter cloacae*	*Escherichia coli*	*Klebsiella* spp.	*Serratia marcescens*	*Acinetobacter* spp.
Penicillines									
Penicillin G	2 (2)	0 (3)	3 (0)	NT	NT	NT	0 (2)	NT	NT
Ampicillin	4 (3)	1 (3)	5 (0)	1 (1)	0 (1)	0 (5)	0 (1)	0 (2)	0 (2)
Ampicillin/Sulbactam	9 (0)	4 (1)	2 (0)	1 (1)	0 (1)	2 (3)	1 (0)	0 (1)	1 (0)
Piperacillin/Tazobactam	6 (0)	3 (1)	3 (0)	1 (1)	5 (0)	3 (0)	3 (0)	0 (1)	1 (0)
Oxacillin	9 (0)	4 (1)	1 (1)	NT	NT	NT	NT	1 (1)	NT
Cephalosporines									
Cefazolin (1) ^1^	4 (0)	3 (1)	1 (0)	NT	NT	NT	NT	0 (1)	NT
Cefuroxim (2) ^1^	11 (0)	6 (1)	5 (0)	0 (2)	NT	3 (2)	NT	0 (1)	1 (0)
Ceftriaxon (3a) ^1^	5 (0)	3 (1)	5 (0)	0 (2)	5 (0)	2 (0)	2 (0)	0 (1)	0 (1)
Ceftazidim (3b) ^1^	2 (0)	1 (1)	3 (0)	0 (1)	5 (0)	3 (1)	3 (0)	1 (1)	1 (2)
Cefepim (4) ^1^	1 (0)	1 (1)	1 (0)	NT	4 (0)	NT	2 (0)	0 (1)	0 (1)
Carbapenemes									
Imipinem	6 (0)	2 (1)	2 (0)	1 (1)	1 (0)	5 (0)	1 (0)	1 (1)	2 (0)
Meropenem	8 (0)	3 (1)	2 (0)	0 (1)	5 (0)	5 (0)	3 (0)	0 (1)	3 (0)
Chinolone									
Ciprofloxacin	7 (0)	5 (1)	2 (1)	1 (0)	5 (0)	4 (1)	3 (0)	1 (0)	3 (0)
Levofloxacin	10 (0)	7 (0)	6 (0)	1 (0)	5 (0)	2 (1)	2 (0)	0 (1)	2 (0)
Glykopeptides									
Vancomycin	9 (0)	5 (0)	4 (0)	2 (0)	NT	NT	NT	1 (0)	NT
Aminoglykosides									
Gentamicin	11 (1)	7 (0)	1 (2)	1 (1)	5 (0)	5 (0)	3 (0)	1 (0)	3 (0)
Oxazolidinone									
Linezolid	8 (0)	5 (0)	2 (0)	2 (0)	NT	NT	NT	NT	NT
Total (n) ^2^	14	7	12	5	5	8	7	3	3

[s (r)] = s, sensitive; r, resistant, spp., species; NT, not tested; ^1^ Cephalosporine 1st to 4th generation; ^2^ Number of times that microorganism were identified in all peritonitis episodes; Numbers may not add up as not all microorganisms were tested for all given antibiotics. Detection of multi-drug-resistant specimen: 1× MRSE; 1× 4MRGN Serratia marcescens; MRSA, VRE, or 3MRGN were not detected.

## 4. Discussion

We report here the first systematic analysis of the pathogens and antibiotic resistance patterns identified in peritonitis patients undergoing ambulatory PD from an outpatient German reference center in the federal state of Brandenburg, Germany. Using retrospective, routinely collected patient data from 2015 to 2022, we found that Gram-positive bacteria were identified more frequently than Gram-negative bacteria and that fungal infections were rare, which is consistent with previous observations in other cohorts [12]. We did not see a systematic trends for a change in the microbiological spectrum in the observational timeframe. The bacterial pathogens were adequately sensitive to the ISPD guideline-recommended antibiotics.

As suggested by the ISPD in 2022, the overall peritonitis rate should be no more than 0.40 (down from 0.5) episodes per year, which was 0.21 for our center in the last year of this study [4]. In our center, we implemented an ongoing quality improvement program for PD, specifically regarding the training and retraining of patients and nurses regarding emerging PD issues and infection prevention [2]. Hence, with increasing patient numbers, the center peritonitis rate was declining, which is generally seen as a reflection of increasing competence and specialization of a PD center [13].

The finding that Gram-positive organisms, especially *Staphylococcus aureus*, *Staphylococcus epidermis*, and streptococci representing the most common pathogens was in keeping with studies from Stuttgart (Germany) [12], Heraklion (Greece) [14], and Amsterdam (The Netherlands) [15].

Clinically, most peritonitis is transmitted by touch resembling common skin and mucosal flora from the nasopharynx, and because hygiene protocols such as the non-touch technique, hand disinfection, or the wearing of face masks are not followed sufficiently. It is of interest to note that according to a general consent of outpatient care providers in Germany, our patients are not required to apply daily mupirocin to the exit site.

We found that in 2022, there was a predominant Gram-negative pathogen sampling in peritonitis. It is unclear if this is an initial indication of a trend change in the bacterial spectrum or an outlier phenomenon, as this year was the last of the study. Due to the ongoing COVID-19 pandemic, the reduction in Gram-positive findings might be secondary to the increased practice of wearing facemasks. In a PD population in Suzhou (China), the proportion of Gram-negative bacteria causing peritonitis increased from 0% to 26.15% over a period of 10 years [16]. Any concern for a trend in this direction should be carefully monitored because PD patients with Gram-negative peritonitis have been found to have lower cure rates, increased severity of symptoms, higher rates of hospitalization, and an increased likelihood to be transferred to hemodialysis [17,18]. Yet, as opposed to Zeng et al. [16], regarding the overall observational timeframe, we did not see systematic changes or trends in the detection rate of microorganism categories.

Unlike the reports from Stuttgart and Amsterdam, *Pseudomonas aeruginosa* was not found to be causative in any of our cases [12,15]. Furthermore, unlike the situation in Stuttgart, we did not find relevant proportions of bacteria that were resistant to multiple antibiotics, specifically MRSA or VRE or mycobacteria [12]. However, we identified in one case solely a sample of methicillin-resistant *Staphylococcus epidermidis* (MRSE), which was treated using vancomycin. In one sample, we detected a 4MRGN *Serratia marcescens*. Although the local bacterial spectra are comparable between our study and the Stuttgart population in terms of the distribution of the Gram-positive and Gram-negative bacteria, the distribution of the individual species and their resistances are different [12]. This may reflect the difference in patient proportion who are treated as inpatients versus outpatients This hypothesis should be considered in future studies. As in most centers in Europe, the US, and increasingly in other regions, APD was the modality most often used by our PD patients [19]. More than 61% of our PD patients used APD, as opposed to approximately 30% who used CAPD. This is noteworthy because some studies suggest that APD patients have a lower risk of peritonitis since they usually only perform one connect and one disconnect per day. In contrast, CAPD generally use up to four dialysis bags per 24 h period, consequently leading to multiple connecting and disconnecting procedures a day, potentially increasing the risk for contamination [20].

Peritonitis due to *Candida* spp. is rare. In our cohort, it was found in one patient with immunosuppression, and in one who was treated with antibiotics for prolonged periods suffering from diabetic foot syndrome and osteomyelitis. *Candida* spp. preferentially adheres to the plastic surfaces of catheters and implants and thus leads to nosocomial candidiasis. This was potentially the cause of our center’s third fungal peritonitis. This patient had two relapses of *Brevibacterium caseii* peritonitis, and even though thorough investigation was performed, no reservoir could be identified for the bacterium. After two cycles of antibiotic therapy, the patient’s condition worsened due to fungal peritonitis, which necessitated removal of the catheter. The patient was able to resume PD with a new PD catheter that was inserted after three months. In patients at risk, prophylactic application of antifungal medication should be considered [21].

Since 2019, we have usually started the antibiotic therapy with cefepime as opposed to cefazolin and ceftazidime; both regimens are in keeping with the ISPD 2022 guidelines [4]. The antibiotic regimen was then subsequently adjusted as necessary after pathogen identification and resistance assessment [4]. IP administration of cefepime is not inferior to the IP administration of the cefazolin and ceftazidime combination [22]. As a fourth-generation cephalosporin, cefepime is a broad-spectrum antibiotic with activity against both Gram-positive and Gram-negative bacteria, as well as against *Pseudomonas aeruginosa*, which is often resistant to a wide range of antibiotics [23]. IP therapy is recommended, as well as the preferred route of administration [4], and cefazolin, ceftazidime, and cefepime are safe and stable for IP application using icodextrin [24].

Acquired resistance to cefepime is generally found in multi-resistant bacteria and in *Staphylococcus hominis* or *Staphylococcus haemolyticus*. Some of these pathogens were in the local bacterial spectrum, but an unfavorable range of resistances were not identified. The sampling frequency of *Staphylococcus aureus*, however, bears individual risk of biofilming, irrespective of antibiotic resistance [25].

Additionally, we recognized the resistance profile of identified *Serratia marcescens* samples that have the ability of to produce extended-spectrum β-lactamases (ESBL) after acquiring the corresponding plasmids [26], and the innate resistance of Enterococcaceae to cephalosporins during antibiotic susceptibility testing. The association of *Enterococcus* spp. with healthcare setting-acquired infections and with a propensity to multi-drug resistance is highly relevant [11]. Specifically, in the event of an initial non-response to treatment with cefazolin and ceftazidime and in patients who have an increased risk of having enterococci, such as in microbial transmural migration from the gastrointestinal tract, vancomycin should be considered to cover the Gram-positive spectrum, including enterococci (e.g., those with chronic, recurrent diverticulitis).

Still, other empiric antibiotic therapies are possible, often combining vancomycin for Gram-positive coverage with ceftazidime or gentamicin for Gram-negative coverage [4]. Specifically, if daily IP treatment is not an option, then vancomycin administered IP every other day in combination with oral ciprofloxacin may be considered.

However, the systematic use of vancomycin in our center is not considered since there is a risk of over- and underdosing that may affect outcome and residual renal function [27,28]. This is of particular relevance as in our outpatient setting, vancomycin level tests are not available on a daily result basis.

This observational study is part of a quality improvement program and so used routinely collected health data extracted from medical records. As a limitation, not all incidents were described in similar detail and there were data missing for 22 peritonitis episodes, and pathogen identification was missing for some patients who underwent treatment in outside hospitals. Accordingly, this study is not intended to provide transferrable clinical value; rather, it was meant to resolve systematic uncertainty regarding our outpatient resistance profiles and to address the empiric selection of calculated therapy at our center. However, the percentage of culture-negative episodes was just 8% (including those from hospitalized patients), suggesting good overall diagnostic standards. Still, the number of patients and peritonitis episodes was limited, which makes the study more descriptive and less informative on a statistical level, specifically regarding the power of the regression analyses performed for trend analyses and regarding widened confidence intervals observed at the beginning and end of the study period which reflect known edge effects in longitudinal modeling. The model’s trend estimates rely on borrowing information from adjacent time points; at the dataset boundaries, the absence of neighbors on one side limits this capability, leading to reduced statistical power and increased estimation uncertainty reflected by greater variance estimates. Our study is retrospective by design and thus limited to the reported outcome measures. Despite these issues, the datasets were sufficient to enable a comprehensive analysis to systematically define the range of infecting organisms causing PD-associated peritonitis and their corresponding antimicrobial resistances.

Variations in pathogen spectra, as we see in our population in comparison to other centers, can result from regional differences, patient-specific factors (such as lifestyle, education, and pet ownership), patient training, center quality standards, and the proportion of inpatient vs. outpatient-acquired peritonitis [29]. Such factors can change; therefore, continued microbiological surveillance and systematic analysis of resistance profiles are justified.

Since antibiotic resistance is currently not a systematic issue and given that the center-specific peritonitis rate has declined, a change in our choice of initial therapy is not currently warranted. If continued, monitoring shows different resistances emerging, then this will need to be addressed. Looking ahead, our data also highlight the importance of careful patient selection for PD to ensure a favorable balance between the benefits of the modality and the risk of peritonitis.

## 5. Conclusions

The spectrum of bacterial pathogens found in peritonitis patients undergoing PD and the respective antibiotic resistance patterns of these bacteria were analyzed and systematically described for an outpatient, northeastern German dialysis center, acknowledging the shortcomings of the study design. One case of multi-resistant *Staphylococcus epidermidis* and one multi-resistant Gram-negative pathogen were identified. Considering the identified Gram-positive spectrum and recognizing the potential side effects of vancomycin, cefepime remains a viable choice of initial therapy for most patients at presentation. When enterococci are strongly suspected or when no clinical and laboratory improvements are seen, the treatment regimen should be changed appropriately.

## Figures and Tables

**Table 1:** Demographic and Clinical Characteristics of Patients and Peritonitis Episodes.

Total PD patients during timeframe, N	129
Most recent PD modality (APD:CAPD:IPD)	79:39:11
Sex (Male; Female)	82; 47
Age at PD initiation, Median (25–75 CI), years	69 (55–78)
Age at first peritonitis episode, Median (25–75 CI), years	73 (62–79)
Time from PD initiation to first peritonitis episode, Median (25–75 CI), months	13 (4–24)
Relapsing peritonits, N (%)	3 (3.75)
Recurrent peritonitis, N (%)	3 (3.75)
Repeat peritonits, N (%)	8 (10.0)
Tunnel affection, N (%)	6 (7.5)
Peritonitis associated modality switch, N (%) *	14 (17.5)
Died in hospital related to peritonitis, N (%)	2 (2.5)

PD = peritoneal dialysis; APD = automated peritoneal dialysis; CAPD = continuous ambulatory peritoneal dialysis; IPD = intermittent peritoneal dialysis (performed in center). Numbers denote Median (1st–3rd quartile) or N (%) as appropriate. * Combined temporal and permanent.

**Table 2:** Etiology of microorganism regarding the peritonitis episodes from 2015 till 2022.

Microorganism	N (%)	Family	Potential Origin
Gram-Positives	56 (56.57)		
*Staphylococcus aureus*	14 (14.14)	*Staphylococcaceae*	upper respiratory tract and skin microbiota
*Staphylococcus epidermidis*	7 (7.07)	*Staphylococcaceae*	typically skin microbiota, less commonly the mucosal microbiota
*Staphylococcus haemolyticus*	6 (6.06)	*Staphylococcaceae*	skin microbiota
*Enterococcus faecialis*	4 (4.04)	*Enterococcaceae*	physiologic gastrointestinal tracts microbiota
*Streptococcus mitis*	4 (4.04)	*Streptococcaceae*	human mouth, throat, and upper respiratory tract microbiota
*Coagulase negative Staphylococci*	2 (2.02)	*Staphylococcaceae*	physiologic skin flora
*Staphylococcus capitis*	2 (2.02)	*Staphylococcaceae*	skin and nasal microbiota
*Streptococcus agalacitae*	2 (2.02)	*Streptococcaceae*	gastrointestinal and genitourinary tract microbiota
*Streptococcus dysgalactiae* ssp. *equisimilis*	2 (2.02)	*Streptococcaceae*	gastroinetstinal and genital tract, less commonly the skin flora.
*Streptococcus salivarius*	2 (2.02)	*Streptococcaceae*	the oral cavity and upper respiratory tract
*Actinomyces neuii*	1 (1.01)	*Actinomycetaceae*	vaginal microbiota
*Bacillus cereus*	1 (1.01)	*Bacillaceae*	found in earth and soil
*Brevibacterium casei*	1 (1.01)	*Brevibacteriaceae*	raw milk and cheese manufacturing
*Coyrnebacterium* spp.	1 (1.01)	*Corynebacteriaceae*	upper respiratory tract and skin microbiota
*Enterococcus* spp.	1 (1.01)	*Enterococcaceae*	gastrointestinal and genitourinary tract microbiota
*Listeria monozytogenes*	1 (1.01)	*Listeriaceae*	gastrointestinal, contaminated drinking water or food
*Micrococcus luteus*	1 (1.01)	*Micrococcaceae*	found in soil, dust, water and air, and as part of the microbiota of the mammalian skin
*Norcadia*	1 (1.01)	*Nocardiaceae*	oral microflora
*Staphylococcus hominis*	1 (1.01)	*Staphylococcaceae*	human and animal skin
*Streptococcus pneumoniae*	1 (1.01)	*Streptococcaceae*	respiratory tract, sinuses, and nasal cavity
*Streptococcus pyogenes*	1 (1.01)	*Streptococcaceae*	skin microbiota
Gram-negatives	31 (31.31)		
*Escherichia coli*	9 (9.09)	*Enterobacteriaceae*	gastrointestinal and genitourinary tract
*Enterobacter cloacae*	5 (5.05)	*Enterobacteriaceae*	gastrointestinal microbiota
*Klebsiella oxytoca*	4 (4.04)	*Enterobacteriaceae*	opportunistic and gastrointestinal origin
*Serratia marcescens*	3 (3.03)	*Yersiniaceae*	ubiquitous in soil, water, animals and plants
*Acinetobacter baumanii*	2 (2.02)	*Moraxellaceae*	hospital environments; environmental soil and water samples
*Klebsiella pneumoniae*	3 (3.03)	*Enterobacteriaceae*	mouth, skin, and intestinal microbiota
*Acinetobacter pittii*	1 (1.01)	*Moraxellaceae*	skin and upper respiratory tract
*Bacteroides uniforms*	1 (1.01)	*Bacteroidaceae*	lower intestinal tract microbiota
*Pantonea agglomerans*	1 (1.01)	*Enterobacteriaceae*	commonly present on plant surfaces and animal or human feces
*Pasteurella multocida*	1 (1.01)	*Pasteurellaceae*	domestic cats and dogs normal respiratory microbiota
*Raoultella (Klebsiella) planticola*	1 (1.01)	*Enterobacteriaceae*	ubiquitous, may colonize plants, water, animals and humans
Fungi	3 (3.03)		
*Candida parapsilosis*	2 (2.02)	*Saccharomycetacea*	hospital environments; catheter surfaces
*Candida* spp.	1 (1.01)	*Saccharomycetaceae*	hospital environments; catheter surfaces
Other	9 (9.09)		
*culture-negative*	8 (8.08)	–	–
*no data record*	1 (1.01)	–	–

## Data Availability

The datasets generated and/or analyzed during the current study are not publicly available due to restrictions specified in the study protocol but are available from the corresponding author on reasonable request.

## Supplementary Materials

The following supporting information can be downloaded at: https://www.mdpi.com/article/10.3390/idr17030049/s1, Supplementary Material S1: RECORD-checklist; Table S1: Yearly sampling of microorganism, Table S2: Peritonitis associated modality drop out; Figure S1: Sampled etiology spectrum 2015–2022, Figure S2: Merged illustration of manuscript Figure 3a,b, Figure S3: Alternative trend modelling.

## List of Abbreviations

The following abbreviations are used in this manuscript:

APD	Automated peritoneal dialysis
CAPD	Continuous ambulatory peritoneal dialysis
ISPD	International Society for Peritoneal Dialysis
IP	Intraperitoneal
MRSA	Methicillin-resistant *Staphylococcus aureus*
MRSE	Methicillin-resistant *Staphylococcus epidermidis*
MRGN	Multi-resistant Gram-negative pathogens
PD	Peritoneal dialysis
spp.	Species
VRE	Vancomycin-resistant enterococci
