# Supplementary material for: Microbiological Surveillance and Antimicrobial Susceptibility Observations on Peritoneal Dialysis-Associated Peritonitis in an Outpatient German Reference Center
Source: Infect Dis Rep. 2025 May 3;17(3):49. doi: 10.3390/idr17030049 (PMC12101265; doi:10.3390/idr17030049)
Supplement: Supplementary file 1 [file idr-17-00049-s001.zip › idr-3511713-supplementary.pdf]

Table S1. Yearly sampling of microorganisms

Microorganism	2015	% of 2015	2016	% of 2016	2017	% of 2017	2018	% of 2018	2019	% of 2019	2020	% of 2020	2021	% of 2021	2022	% of 2022
**Gram-positives**																
**Staphylococcus aureus**	3	20,0	2	22,2	3	27,3			2	13,3	2	11,8	2	18,2		
**Staphylococcus epidermidis**	1	6,7	1	11,1	1	9,1	1	7,7			1	5,9	2	18,2		
**Staphylococcus haemolyticus**	3	20,0					1	7,7					1	9,1	1	12,5
**Enterococcus faecialis**	2	13,3			1	9,1							1	9,1		
**Streptococcus mitis**			1	11,1					1	6,7	2	11,8				
**Coagulase negative Staphylococci**	1	6,7											1	9,1		
**Staphylococcus capitis**									1	6,7	1	5,9				
**Streptococcus agalacitae**									1	6,7	1	5,9				
**Streptococcus dysgalactiae ssp equisimilis**									1	6,7			1	9,1		
**Streptococcus salivarius**			1	11,1							1	5,9				
**Actinomyces neuii**													1	9,1		
**Bacillus cereus**									1	6,7						
**Brevibacterium casei**									1	6,7						
**Coyrnebacterium spp**									1	6,7						
**Enterococcus spp.**					1	9,1										
**Listeria monozytogenes**			1	11,1												
**Micrococcus luteus**					1	9,1										
**Norcadia**							1	7,7								
**Staphylococcus hominis**							1	7,7								
**Streptococcus pneumoniae**					1	9,1										
**Streptococcus pyogenes**											1	5,9				

Table S1. continuation

Microorganism	2015	% of 2015	2016	% of 2016	2017	% of 2017	2018	% of 2018	2019	% of 2019	2020	% of 2020	2021	% of 2021	2022	% of 2022
**Gram-negatives**																
**Escherichia coli**	2	13,3	1	11,1	1	9,1	1	7,7	1	6,7	1	5,9			2	25
**Enterobacter cloacae**	1	6,7					2	15,4	2	13,3						
**Klebsiella oxytoca**											2	11,8	1	9,1	1	12,5
**Serratia marcescens**									2	13,3					1	12,5
**Acinetobacter baumanii**							1	7,7			1	5,9				
**Klebsiella pneumoniae**					1	9,1									2	25
**Acinetobacter pittii**											1	5,9				
**Bacteroides uniforms**	1	6,7														
**Pantonea agglomerans**			1	11,1												
**Pasteurella multocida**					1	9,1										
**Raoultella (Klebsiella) planticola**							1	7,7								
**Fungi**																
**Candida parapsilosis**	1	6,7									1	5,9				
**Candida spp**							1	7,7								
**Other**																
**culture-negative**							3	23,1	1	6,7	2	11,8	1	9,1	1	12,5
**no data record**			1	11,1												

Table S2. Peritonitis associated modality drop out

Episode	Number of microorganism	Species
1	2	Streptococcus dysgalactiae ssp equisimilis<br>Staphylococcus aureus
2	1	Streptococcus mitis
3	2	Staphylococcus epidermidis<br>Candida parapsilosis
4	1	Klebsiella oxytoca
5	1	Staphylococcus aureus
6	1	Staphylococcus aureus
7	1	Enterobacter cloacae
8	1	Staphylococcus haemolyticus
9	3	Klesiella oxytoca<br>Enterococcus faecialis<br>Coagulase negative Staphylococci
10	2	Staphylococcus capitis<br>Bacillus cereus
11	1	Coagulase negative Staphylococci
12	1	Escherichia coli
13	1	Candida parapsilosis
14	1	Acinetobacter baumanii
15	1	Enterobacter cloacae

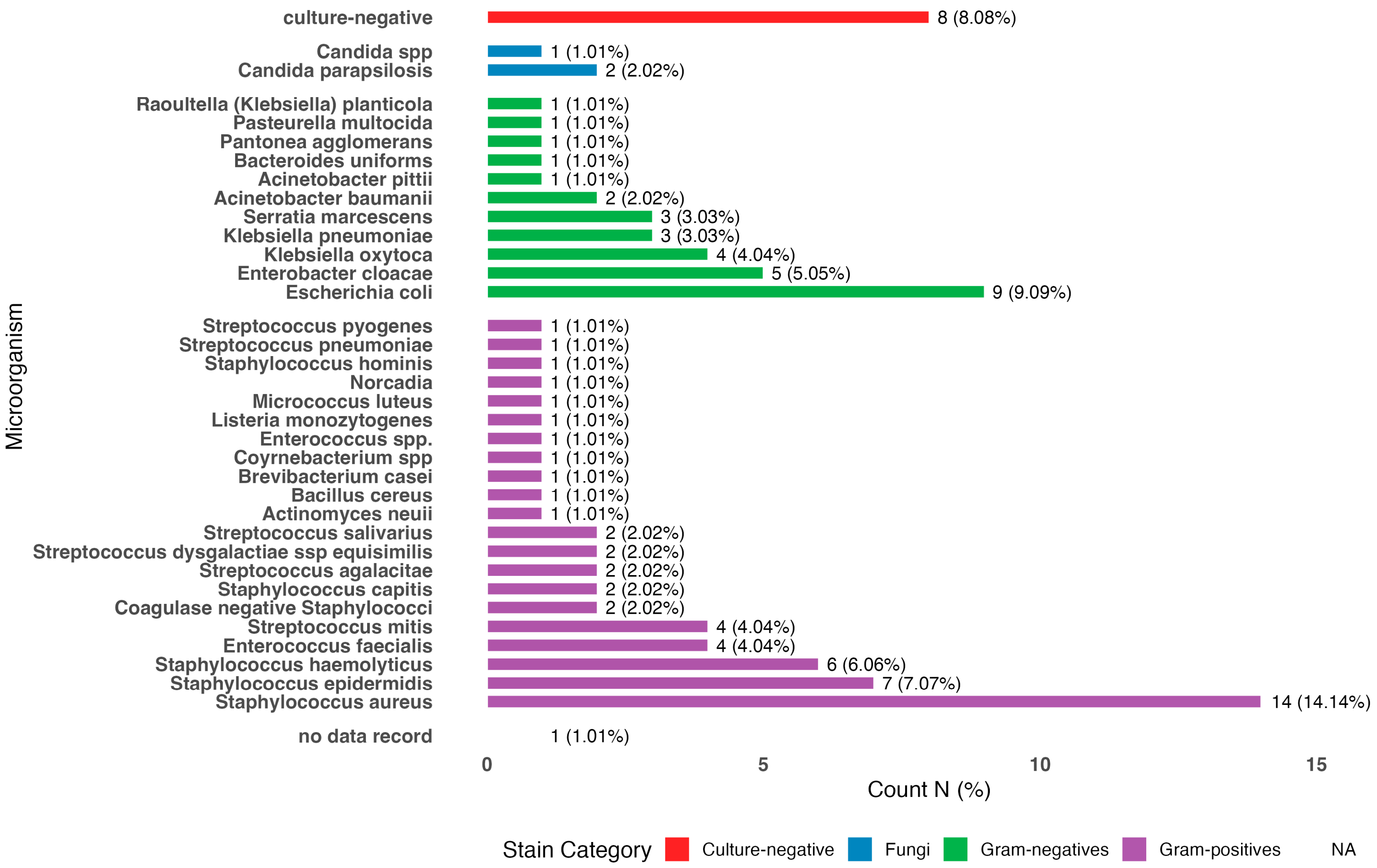

Figure S1. Sampled etiology spectrum 2015-2022

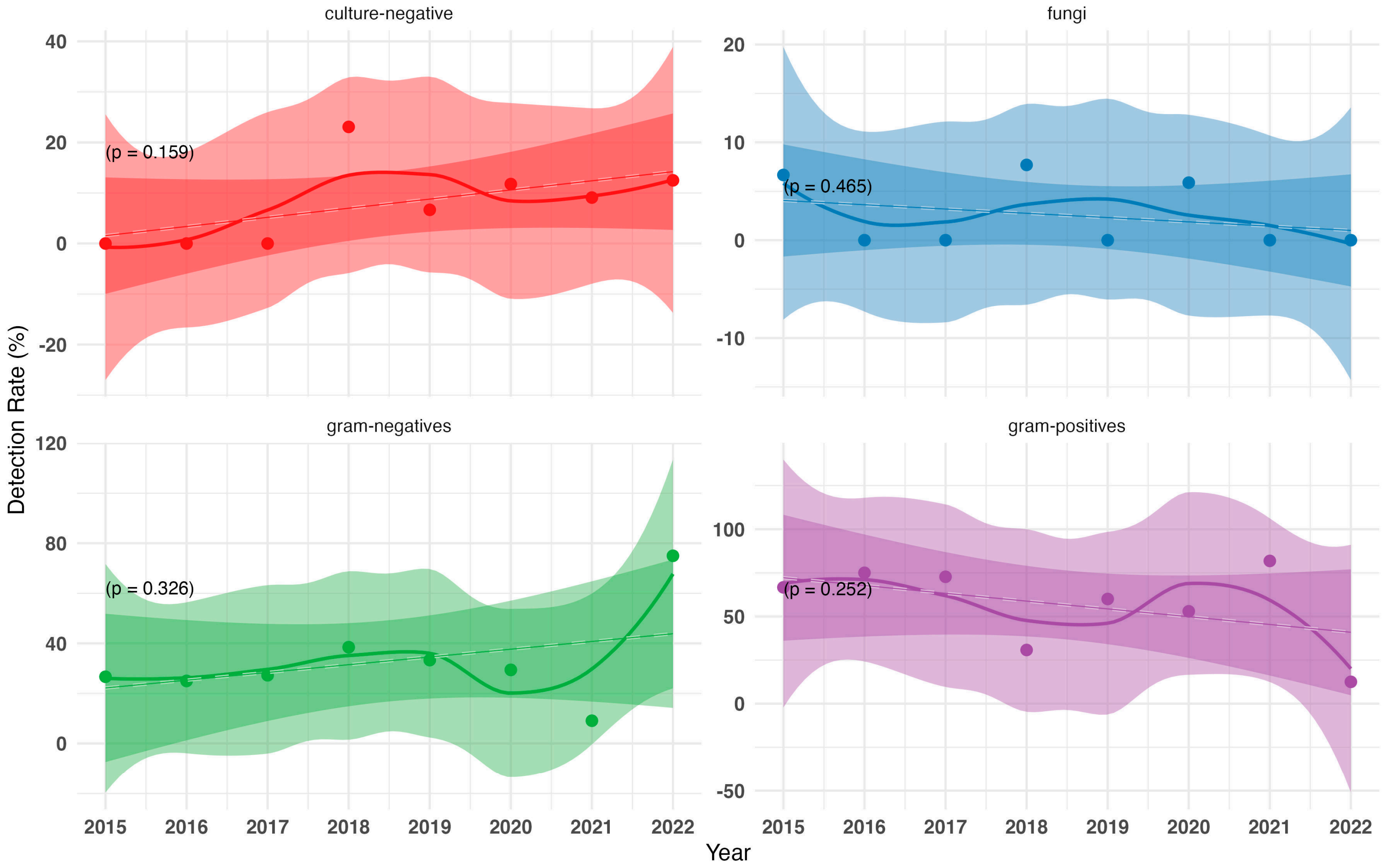

Figure S2. Merged illustration of manuscript **Figure 3a** and **Figure 3b** illustrating Locally Estimated Scatterplot Smoothing (LOESS) and Ordinary Least Squares (OLS) regression to model trends in the detection of microbiological spectrum samples.

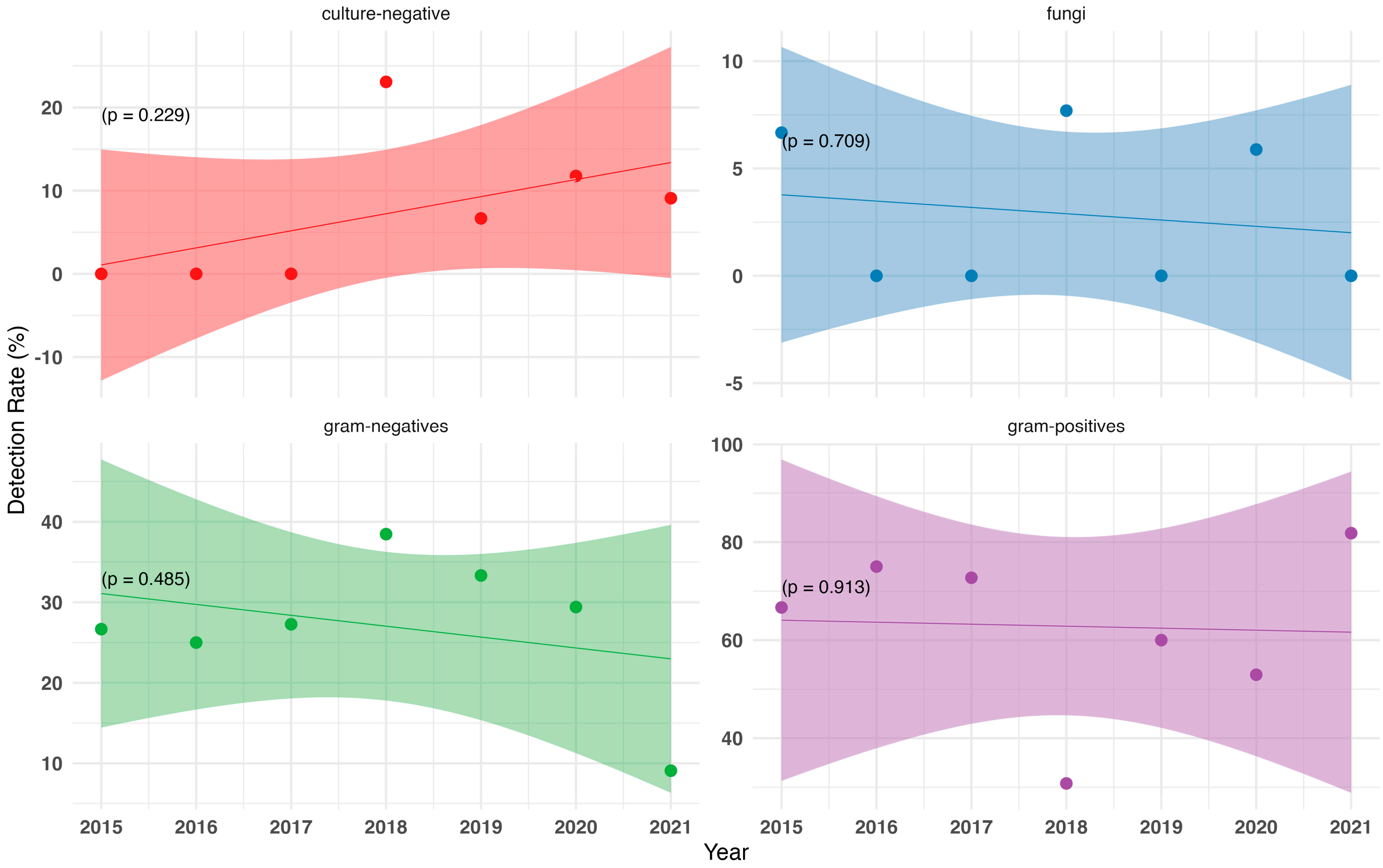

Figure S3. Alternative trend modelling.

Suggesting that the high gram-negative count in 2022 was an outlier phenomenon, we used the OLS model excluding year 2022. Given this hypothetical data there was no indication of an increase in gram-negative bacteria (P=0.485) and an almost horizontal trend line for the gram-positive bacteria (P=0.913).
